# From Optical to AI-Driven Markerless Motion Capture in Motor Learning and Rehabilitation

**DOI:** 10.3390/bioengineering13070776

**Published:** 2026-07-03

**Authors:** Panagiotis Georganakis, Konstantinos Spinthiropoulos, Konstantinos Panitsidis, Dimitrios Parris, Vasiliki Gerodimou

**Affiliations:** Department of Management Science and Technology, School of Economics, University of Western Macedonia, 50100 Kozani, Greece; kspinthiropoulos@uowm.gr (K.S.); kpanytsidis@uowm.gr (K.P.); mpu00517@uowm.gr (V.G.)

**Keywords:** markerless motion capture, human biomechanics, 3D pose estimation, deep learning, motor learning, clinical rehabilitation, healthcare

## Abstract

Traditional biomechanical analysis is constrained by high capital costs and the physical limitations imposed by markers, posing significant barriers to clinical adoption. This review evaluates the emergence of artificial intelligence (AI)-based markerless motion capture (MMC) as a transformative approach for democratizing movement science in clinical rehabilitation. The discussion outlines the progression from legacy geometric visual hulls to advanced deep learning architectures, with particular focus on YOLO-based two-dimensional detection and spatio-temporal transformer models for three-dimensional pose estimation. Evidence indicates that multi-camera MMC frameworks achieve research-grade positional accuracy (16–34 mm Mean Per-Joint Position Error—MPJPE), while monocular systems provide sufficient sensitivity (82–88%) for longitudinal monitoring of geriatric fall risk and stroke recovery. While challenges persist in achieving precise axial rotation measurement, integrating real-time signal refinement enables objective and ecologically valid assessments in community-based healthcare settings. This technological advancement redefines movement analysis, shifting it from a laboratory-bound procedure to a widely accessible and interoperable diagnostic tool.

## 1. Introduction

The quantitative assessment of human movement is changing rapidly due to advancements in artificial intelligence (AI) and real-time computer vision [1]. Human movement analysis (the measurement and interpretation of how people move, frequently used in clinical diagnosis and rehabilitation) has historically relied on optoelectronic marker-based systems, which use light-emitting markers attached to the body to track motion and analyze mechanical principles of movement [2]. While these systems remain highly accurate and are considered the gold standard, they are expensive ($150,000–$500,000+), require complex infrastructure, and involve attaching markers or sensors to subjects, a process that could restrict natural movement [1,3]. This review examines how AI-driven markerless motion capture (MMC), which uses cameras and computer algorithms to analyze movement without physical markers, enables wider access to movement science, supporting its shift from elite research labs to practical clinical settings in local communities [1].

### 1.1. The Evolution of Motion Capture Paradigms

The trajectory of human locomotion analysis can be bifurcated into the Historical Digital Era (1880s–1990s) and the Markerless Transition (2000s–2010s), leading into the current AI-driven era [1]. Early pioneers like Muybridge and Marey utilized sequentially triggered photography to resolve biomechanical questions invisible to the human eye, establishing the first temporal–spatial datasets. The 1970s and 1980s redefined the field with the advent of optoelectronic systems that track infrared-reflective markers to automate the reconstruction of 3D joint centers [2]. However, these systems remain limited by subject encumbrance and the pervasive soft-tissue artifact (STA), where skin-mounted markers shift relative to the underlying bone, introducing kinematic errors often exceeding 10 mm and 10° [4].

Between 2000 and 2010, a critical transitional era began for markerless motion analysis [3]. In this period, scientists reduced reliance on physical skin markers and instead used model-based methods. These methods used visual hulls, which are 3D shapes created by combining outlines (silhouettes) of the body from several cameras [3,5]. This phase helped develop mathematical tools, such as Spatio-Temporal Nonlinear Dimension Reduction (which reduces complex movement data into simpler forms), and algorithms, such as Annealed Particle Filtering (a technique for estimating probable joint positions). These innovations provided new ways to reconstruct joint movement in the lab without the need for physical markers [6,7].

By framing the 2000–2010 period as the transitional phase, the manuscript clarifies that this decade is not merely a historical background but an era of geometric validation that preceded the semantic validation of current deep learning architectures [1,8].

### 1.2. Analytical Framework: The “Democratization” of Biomechanics

Rather than relying solely on descriptive terminology, “democratization” is defined here as the systematic removal of socio-technical barriers to movement science across three rigorous dimensions, detailed below.

• Economic Accessibility: The transition from proprietary, high-cost laboratory hardware to consumer-grade RGB cameras and open-source deep learning frameworks [1,9,10].
• Operational Decoupling: The elimination of requiring specialized technicians and 60 min subject preparation times, enabling on-demand assessment in various clinical settings [1,11].
• Ecological Validity: The capacity to perform movement analysis in street clothing within the subject’s natural environment, thereby mitigating the psychological bias and behavioral modifications associated with laboratory suites [1,12].

While MMC technology is used in sports coaching and motor learning—domains that often tolerate higher error margins for qualitative technique feedback [1]—this review focuses on clinical rehabilitation, where the risk of error is highest and longitudinal stability is paramount for patient safety and effective fall risk prediction [1,9].

### 1.3. Emergence of AI and Spatio-Temporal Lifting

Modern MMC leverages convolutional neural networks (CNNs), which are machine learning models designed to process images, and transformer architectures, advanced AI models for recognizing patterns in data, to identify anatomical keypoints (specific joint locations) directly from standard video data [8]. However, monocular RGB data (color video from a single camera) is fundamentally ambiguous, as a single 2D projection can represent an infinite number of 3D configurations. This bioengineering challenge is called depth ambiguity [1]. Contemporary solutions address this by analyzing changes across multiple video frames (temporal patterns) and by applying biomechanical priors, such as the assumption that bone lengths remain constant [13,14,15]. By identifying semantic joint centers (algorithmically estimated joint locations) rather than physical markers attached to the body, AI-MMC may theoretically avoid the errors inherent in soft tissue artifact (STA), assuming these models are validated against bone-anchored gold standards [1,16].

The clinical value of markerless kinematic measurement is closely coupled to the broader field of rehabilitation robotics, which provides several of the control- and estimation-theoretic building blocks upon which closed-loop, vision-guided rehabilitation depends. Trajectory-planning strategies for robotic manipulators—for example, methods combining dynamic movement primitives (DMP) with artificial potential fields (APF) for the reduction in fractures using parallel robots—illustrate how anatomically constrained motion paths can be generated and adapted in real time [17]. In lower-limb rehabilitation robots, accurate and responsive estimation of human–robot interaction torque is essential for safe, compliant assistance, and high-accuracy estimators with strong tracking ability have been developed for exactly this purpose [18]. Likewise, finite-time observer-based variable-impedance control of cable-driven continuum manipulators demonstrates the kind of robust state estimation and impedance regulation that would allow markerless kinematic feedback to be safely incorporated into assistive devices [19]. Positioning AI-MMC alongside these control and estimation advances clarifies how objective, marker-free movement measurement can ultimately close the perception–action loop in next-generation rehabilitation systems.

### 1.4. Research Questions (RQs)

Within this context, the following central research questions provide the organizational framework for the evidence synthesis and guide the ensuing discussion throughout the review.

• RQ1: How does the positional fidelity (MPJPE) of contemporary AI lifting architectures compare across monocular and multi-view configurations in clinical gait assessment?
• RQ2: To what extent do environmental variables, specifically clothing conditions, degrade the kinematic validity of markerless systems in rehabilitative settings?
• RQ3: Can AI-based longitudinal monitoring identify cumulative changes in movement variability with sufficient sensitivity to predict clinical events like geriatric falls?

## 2. Materials and Methods

### 2.1. Search Strategy

To ensure the evidentiary weight and reproducibility of this review, a search was executed across Scopus, PubMed, and the ACM Digital Library, among others. The search was designed to capture the evolution of movement informatics from early geometric models to contemporary deep learning architectures [1].

For full reproducibility, the search combined three concept blocks with Boolean operators, using OR within each block and AND between blocks, applied to titles, abstracts, and keywords. Block 1 (technology): (“markerless motion capture” OR “marker-free motion capture” OR “pose estimation” OR “markerless” OR “OpenPose” OR “DeepLabCut” OR “Theia3D” OR “YOLO”). Block 2 (method/domain): (“deep learning” OR “convolutional neural network” OR “transformer” OR “3D pose” OR “kinematics” OR “gait analysis” OR “biomechanics”). Block 3 (application): (“rehabilitation” OR “clinical” OR “stroke” OR “cerebral palsy” OR “fall risk” OR “geriatric” OR “motor learning”). The representative full query was therefore of the following form: (Block 1) AND (Block 2) AND (Block 3). Searches were limited to peer-reviewed journal articles, conference proceedings, and books published between 2000 and 2025 and written in English, and database-specific syntax (e.g., TITLE-ABS-KEY in Scopus, MeSH terms in PubMed) was adapted accordingly. The last search was run in 2025, and reference lists of key reviews were hand-searched to identify additional records.

The review statement period encompasses 2000–2025; as established in Section 1.1, the 2000–2010 decade serves as the transitional era of silhouette-based geometric validation, while the post-2010 era represents the Active Deep Learning Era of semantic joint localization [1,20].

#### Inclusion and Quality Appraisal

A total of 93 full-text articles were evaluated with a focus on clinical rehabilitation and rehabilitative robotics, and 46 studies were synthesized for the final analysis. This selection also incorporates 18 high-quality clinical studies identified in the landmark secondary synthesis by Knippenberg et al., which utilized the Van Tulder Scale to establish a mean quality score of 8.06 ± 3.67, indicating high scientific reliability [18].

**Figure 1:**
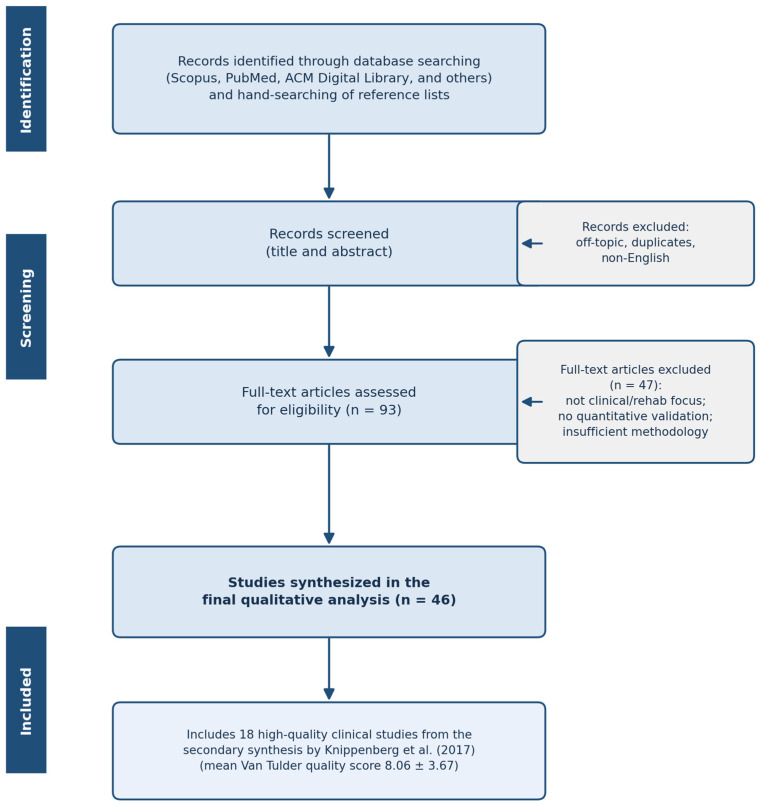
PRISMA 2020 flow diagram of the study identification, screening, and inclusion process (adapted from Knippenberg et al. [21]).

Studies were eligible for inclusion if they (i) applied or validated a markerless or AI-based motion-capture/pose-estimation method, (ii) reported quantitative performance (e.g., MPJPE, joint-angle error, sensitivity/specificity) or addressed a clinically relevant rehabilitation, motor-learning, or fall-risk application, (iii) were peer-reviewed journal articles, conference proceedings, or scholarly books, and (iv) were published between 2000 and 2025 in English. Studies were excluded if they (i) were unrelated to human movement analysis or to the clinical/rehabilitation focus, (ii) used exclusively marker-based or wearable-only systems without a markerless comparison, (iii) provided no quantitative validation or methodological detail, (iv) were duplicates, editorials, abstracts without full text, or non-English records, or (v) were of insufficient methodological quality. The screening and selection process is summarized in the PRISMA 2020 flow diagram (Figure 1).

### 2.2. The Informatics Pipeline: 2D Detection and 3D Lifting

A primary factor enabling wider access to clinical movement analysis is the transition from multi-stage, bottom-up models to efficient, end-to-end deep learning architectures, which underpin the informatics pipeline described in the subsequent sections [22].

#### 2.2.1. 2D Anatomical Keypoint Detection

Contemporary AI systems primarily utilize YOLO (You Only Look Once) architectures for high-speed keypoint detection [8]. Beyond YOLO, OpenPose, and DeepLabCut, Google’s MediaPipe (BlazePose GHUM Holistic) has become a widely adopted state-of-the-art framework, delivering real-time, on-device estimation of 33 body landmarks from a single RGB image and is therefore especially relevant for accessible, smartphone-based clinical deployment [23]. A defining characteristic of these monocular pipelines is that the mapping from 2D image keypoints to 3D coordinates is learned rather than measured: the lifting networks must be trained on very large annotated motion-capture corpora—most prominently Human3.6M, which provides 3.6 million 3D human poses recorded with synchronized marker-based ground truth—so that the model can infer plausible depth from monocular appearance. The scale and diversity of this training data directly bound the accuracy and generalization of the resulting estimators [24]. Standardization of anatomical joint localization is a prerequisite for cross-system validation; the specific keypoint mapping and coordinate nomenclature utilized by these deep learning architectures are comprehensively detailed in Table A1 of Appendix A [25,26].

#### 2.2.2. Transformer-Based 3D Pose Lifting

To resolve the monocular depth ambiguity—where a 2D joint projection can represent an infinite number of 3D configurations—current state-of-the-art models employ transformer architectures such as TCPFormer [13]. These models use self-attention mechanisms to analyze temporal dependencies over a nine-frame window, enforcing biomechanical priors to ensure that joint trajectories remain anatomically plausible [13,27].

### 2.3. Signal Processing and Kinematic Extraction

Raw AI-derived joint coordinates frequently exhibit high-frequency jitter due to fluctuating localization confidence [26]. Two primary signal refinement strategies are examined:

1. **Adaptive Jitter Attenuation:** Real-time jitter attenuation without the introduction of phase lag is achieved via adaptive cutoff frequency modulation; the governing algorithmic logic of the **1-Euro Filter** is provided in Appendix B.2 [28,29].
2. **State–Space Modeling:** Estimation of biomechanical derivatives, such as center-of-mass (CoM) velocity, relies on linear discrete-time state–space models; the mathematical transition logic governing the **Extended Kalman Filter (EKF)** is formalized in Appendix B.3 [28,30].

### 2.4. Biomechanical Validation and Interoperability

The extraction of action-specific kinematics from refined coordinate sets involves rigorous vector-based geometric computation; the definitive mathematical formulas for calculating sagittal plane joint angles (e.g., knee flexion) are presented in Appendix B.1 [1,31]. To facilitate clinical democratization, the resulting kinematic data must be interoperable with modern healthcare systems. A representative JSON schema designed for the seamless integration of AI-MMC data is illustrated in Appendix C [9,32].

**Figure 2:**
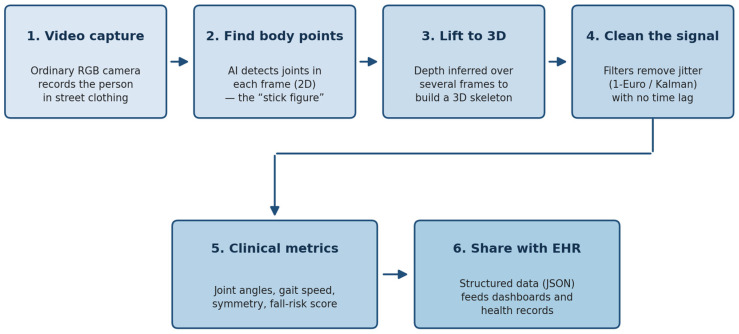
Plain-language workflow of the AI-MMC pipeline, from ordinary video to clinical metrics and health-record integration.

In plain language for the clinical reader, the technical machinery in this section can be read as a simple six-step pipeline that turns ordinary video into clinical numbers, summarized in Figure 2. First, an everyday RGB camera records the patient moving in their own clothing. Second, an AI model finds the body’s joints in every frame, producing a two-dimensional “stick figure.” Third, the model uses information from several consecutive frames to estimate depth and assemble a three-dimensional skeleton. Fourth, smoothing filters—the 1-Euro filter and the Kalman filter—remove the small frame-to-frame shaking (“jitter”) in the tracked points without introducing a noticeable time delay; conceptually, they behave like a noise-canceling step that keeps fast movements sharp while damping random flicker. The mathematical formulas for the joint-angle calculation and for these filters are provided in Appendix B for completeness, but they are not required to interpret the clinical output. Fifth, the cleaned skeleton is converted into familiar clinical metrics such as joint angles, walking speed, gait symmetry, and a fall-risk score. Sixth, these metrics are exported in a structured data format (the JSON schema in Appendix C is simply a standardized “container” that lets the numbers flow automatically into clinical dashboards and electronic health records). Readers who do not need the underlying equations can therefore follow the workflow at the level of these six steps.

## 3. Comparative Analysis of Validation Studies

The empirical synthesis of contemporary validation studies confirms that artificial intelligence (AI)-based markerless motion capture (MMC) has reached an accuracy threshold suitable for clinical deployment [1,28,33]. This section evaluates the quantitative performance of these architectures against established optoelectronic benchmarks, focusing on positional fidelity, rotational sensitivity, and the impact of environmental variables on clinical interpretation [12,34].

For clarity, the evidence in this review is organized around three thematic pillars that map directly onto the research questions stated in Section 1.4. The first pillar, technical validation, concerns measurement accuracy, environmental robustness, and real-time signal filtering, and is addressed in the present section (Section 3.1, Section 3.2, Section 3.4 and Section 3.5; RQ1 and RQ2). The second pillar, clinical validation, concerns performance in neurological and geriatric populations and is addressed in Section 3.3 and developed further in Section 4.3 (RQ3). The third pillar, remaining bottlenecks with actionable solutions—axial rotation, privacy, and regulation—is addressed in the Discussion (Section 4.4, Section 4.5, Section 4.6 and Section 4.7). This thematic structure is used consistently in the Comparative Analysis and the Discussion so that each block of evidence is explicitly linked to the question it answers.

### 3.1. Benchmarking Positional Fidelity: The MPJPE Metric

The primary metric for quantifying pose estimation accuracy in movement science is the Mean Per-Joint Position Error (MPJPE), which identifies the average Euclidean distance between predicted semantic keypoints and ground-truth marker centers [16]. Contemporary multi-camera RGB systems, using deep learning frameworks such as OpenPose, show joint center differences of 16–34 mm during dynamic tasks such as walking and jumping [25,30]. Validation data suggest that nearly 80% of the mean absolute errors (MAEs) in AI-driven joint localization remain below the 30 mm threshold required for research-grade gait analysis [1,16,35,36].

An important caveat when interpreting these aggregate error figures is that accuracy is not isotropic across the three spatial axes. For monocular RGB pipelines, the in-plane (image) coordinates of a landmark are typically estimated with high fidelity, because the network is directly localizing a visible feature in the image; the depth (out-of-plane) coordinate, by contrast, is inferred and is consequently the least accurate dimension, dominating the overall positional error. Reported MPJPE values therefore tend to be driven by depth error rather than by in-image localization error. This anisotropy is precisely the limitation that RGB-D sensing mitigates by measuring depth directly, and it should be borne in mind when comparing systems on a single scalar accuracy metric.

### 3.2. Homogenized Comparative Evaluation of MMC Systems

To address procedural heterogeneity in the existing literature, Table 1 provides a standardized comparison of system performance across hardware configurations and clinical application domains [9,26].

Analytical results indicate that while multi-view configurations are mandatory for calculating center-of-mass (CoM) derivatives in high-velocity activities, monocular systems provide sufficient spatiotemporal resolution for pathology monitoring and fall-risk screening [9,30].

A third sensing category sits between these two extremes and deserves explicit consideration: low-cost RGB-D (RGB-plus-depth) cameras such as the Microsoft Kinect/Azure Kinect and the Intel RealSense family. Unlike purely RGB systems, RGB-D devices directly measure per-pixel depth using structured-light or time-of-flight sensing, so they do not have to solve the ill-posed problem of inferring the depth coordinate from a single 2D image; the third spatial dimension is acquired, rather than learned. This sidesteps the monocular depth-ambiguity that limits RGB-only pipelines, while remaining inexpensive and markerless. Validation studies comparing the Kinect v2 and Azure Kinect against optoelectronic gold standards have reported good agreement for sagittal-plane lower-limb joint angles and spatiotemporal gait parameters, with the Azure Kinect generally outperforming the earlier Kinect v2 [38]. Their principal limitations are a shorter effective operating range, sensitivity to ambient infrared and sunlight, a narrower field of view, and reduced reliability for fine distal segments (e.g., ankle and foot) and transverse-plane rotation. In practice, therefore, RGB-D cameras offer a favorable accuracy-to-cost trade-off for constrained indoor clinical and home settings, whereas RGB-based deep-learning pipelines remain more flexible for unconstrained, in-the-wild capture from existing video. A direct, application-specific comparison of RGB versus RGB-D performance is consequently an important consideration when selecting a system for a given rehabilitation task.

To make these configuration-specific trade-offs concrete, Table 2 consolidates representative positional (MPJPE) and axial-rotation error values for monocular versus multi-view systems across the principal clinical use cases considered in this review, together with the clinically acceptable thresholds reported for each. For research-grade gait analysis, a per-joint error below approximately 30 mm is generally regarded as acceptable, sagittal-plane joint angles are expected to fall within roughly 2–5°, and transverse-plane (axial) rotation remains the weakest dimension, with errors commonly exceeding 10° against a desirable target of below 5–10°.

### 3.3. Clinical Validation in Neurological Populations

The widespread adoption of movement science is most evident in the successful automation of clinical scoring for neurological disorders [1]. In stroke rehabilitation, monocular MMC using DeepLabCut has proven effective for identifying kinematic asymmetries between affected and unaffected limbs, facilitating objective longitudinal assessment [37,39]. Similarly, AI-driven gait reports for children with cerebral palsy now automate parameters such as cadence and peak knee flexion, achieving a level of standardized accuracy that removes the inter-rater variability typical of subjective observation [40,41,42].

Beyond simple feasibility, monocular MMC offers distinct advantages for long-term, home-based rehabilitation monitoring. Because it requires only a single consumer-grade RGB camera and no marker placement or technician supervision, it enables frequent, unsupervised assessments in the patient’s own environment, capturing day-to-day fluctuations in motor performance that single-visit laboratory sessions cannot resolve. This longitudinal density is particularly valuable for tracking limb motor asymmetry, the hallmark of post-stroke and hemiparetic recovery. Typical kinematic indicators used to quantify such asymmetry include the inter-limb symmetry index (the normalized percentage difference between the affected and unaffected limb), the ratio of paretic to non-paretic step length and stance time, bilateral differences in peak knee and hip flexion angles during swing, sagittal-plane range-of-motion ratios, peak angular velocity asymmetry, and temporal asymmetry in gait sub-phase durations. Tracking the convergence of these indices toward unity over successive home sessions provides an objective, interpretable marker of functional recovery and treatment response [37,39].

Regarding diagnostic performance, the 82–88% sensitivity reported for depth-sensor fall-risk screening should be interpreted alongside complementary diagnostic indicators. The same body of work reports specificities in the order of 78–86%, an overall classification accuracy of approximately 80–85%, and areas under the receiver-operating-characteristic curve (AUC) typically between 0.82 and 0.90, with positive and negative predictive values that vary with the baseline fall prevalence of the screened population [9,43]. For a community screening tool, where the goal is to flag at-risk individuals for further clinical assessment rather than to confirm a diagnosis, high sensitivity is the priority, and these values approach—but do not yet uniformly meet—the ≥90% sensitivity often cited as desirable for population-level screening. Accordingly, AI-MMC is currently best positioned as a triage and longitudinal-monitoring instrument that prompts confirmatory evaluation, rather than as a stand-alone diagnostic replacement, and future reports should consistently disclose the full confusion matrix to permit transparent comparison against clinical screening standards.

The application of MMC to children, and in particular to children with cerebral palsy, introduces challenges that go beyond those encountered in adult populations. Most pose-estimation networks are trained predominantly on adult body data, so their learned anatomical priors and segment-length proportions do not transfer cleanly to the smaller stature, larger head-to-body ratio, and distinct limb proportions of pediatric subjects, which can degrade keypoint localization accuracy. Atypical and highly variable movement patterns—spasticity, crouch gait, involuntary movements, toe-walking, and frequent use of assistive devices—fall outside the distribution of typical training datasets and increase the risk of keypoint mis-assignment and tracking failure. Compliance and attention spans are also shorter in children, complicating the capture of clean, repeatable trials. Mitigation strategies include fine-tuning or domain-adapting models on pediatric and clinical datasets, incorporating child-specific anthropometric priors and skeletal scaling, and using temporal smoothing constrained by physiologically plausible pediatric ranges of motion. These considerations underscore that pediatric validation must be conducted separately and should not be inferred from adult performance figures [40,41].

### 3.4. Environmental Robustness and Ecological Validity

A fundamental requirement for biomechanics is the system’s ability to maintain fidelity under non-laboratory conditions, specifically regarding subject attire [12]. Comparative investigations between sport clothing (fitted gear) and street clothing (unrestricted attire) using the Theia3D system revealed mean differences in segment lengths (forearm, thigh, and shank) of only 0.2–0.9 cm [12]. These discrepancies are statistically smaller than the 1–2 cm error typical of marker placement by expert technicians in laboratory settings [12,44,45]. Furthermore, root-mean-square deviations (RMSD) for joint angles between clothing conditions averaged 2.6°, suggesting that “street clothing” does not significantly degrade clinical gait interpretation [12].

Although the clothing experiments summarized above isolate a single variable, real clinical and community deployments expose MMC systems to several additional sources of interference that must be considered when judging ecological robustness. Uneven or low illumination reduces image contrast and the confidence of keypoint detection, increasing jitter and the likelihood of dropped frames; this can be partially mitigated through depth or infrared sensing, high-dynamic-range cameras, and training data augmented with diverse lighting conditions. Partial occlusion—by furniture, assistive devices, loose clothing, or self-occlusion during turning—remains one of the most common failure modes, but occlusion-aware and temporally informed transformer architectures can infer hidden joints from surrounding frames and biomechanical priors, and multi-camera configurations provide redundant viewpoints that recover occluded segments. Complex or cluttered backgrounds raise the risk of false detections, which can be reduced by robust person-segmentation, attention-based region proposals, and background-invariant feature learning. The presence of multiple people in the field of view introduces identity-association and tracking errors; top-down detection with re-identification and multi-object tracking, together with operator-defined regions of interest, helps maintain a stable subject track. Overall, while contemporary MMC systems retain useful accuracy under moderate perturbation, their anti-interference capacity degrades as several of these factors co-occur, so validation protocols should explicitly stress-test lighting, occlusion, background complexity, and crowding, rather than reporting performance only under controlled single-subject conditions [13,27,46].

### 3.5. Real-Time Signal Smoothing and Jitter Attenuation

Raw joint coordinates derived from real-time architectures, such as YOLOv8 or OpenPose, frequently exhibit high-frequency “jitter” [8,29]. The application of the adaptive 1-Euro filter has demonstrated the ability to stabilize joint trajectories in real time without introducing the prohibitive phase lag associated with traditional Butterworth filters [28,29]. In high-speed applications, integrating an Extended Kalman Filter (EKF) reduced velocity errors from 0.943 m/s to 0.257 m/s, bridging the gap between qualitative visualization and quantitative research-grade biomechanics [26,28].

## 4. Discussion

The transition of motion capture from high-capital-expenditure laboratory environments to community-level clinical settings constitutes a fundamental paradigm shift in movement science [1]. Comparative analyses of validation studies indicate that artificial intelligence (AI)-based markerless motion capture (MMC) has achieved a level of positional fidelity that challenges the established gold standard set by optoelectronic marker-based systems [16]. The following discussion systematically addresses the core research questions identified in Section 1.4, evaluating the bioengineering trade-offs inherent in this process of democratization, the algorithmic strategies required to resolve key kinematic ambiguities, and the clinical implications of real-time movement monitoring for accessibility, measurement accuracy, and predictive capacity [47,48].

Consistent with the three-pillar organization introduced in Section 3, the Discussion is structured to move from technical to clinical validation and finally to the remaining bottlenecks and their actionable solutions. Section 4.1 and Section 4.2 consolidate the technical-validation pillar (accuracy–accessibility trade-offs, depth ambiguity, and real-time filtering; RQ1 and RQ2); Section 4.3 develops the clinical-validation pillar (longitudinal monitoring in geriatric and neurological populations, with its ethical dimension; RQ3); and Section 4.4, Section 4.5, Section 4.6 and Section 4.7 address the third pillar, the remaining bottlenecks—evaluation standardization, axial rotation, and a dedicated treatment of future challenges, including fairness, deployment, regulation, and gait-based re-identification. This alignment ensures that each part of the Discussion answers a specific research question posed in Section 1.4.

### 4.1. The Accuracy–Accessibility Trade-Off: Reevaluating the “Gold Standard”

A critical question emerges from the recent literature: does sub-millimeter marker precision inherently equate to superior anatomical accuracy? Historically, optoelectronic systems have been lauded for their precision in tracking physical retro-reflective markers [2]. However, the bioengineering community increasingly acknowledges the soft tissue artifact (STA) as a pervasive source of error [4]. STA occurs when muscle contraction and inertial vibration cause skin markers to shift relative to the underlying bone, introducing errors that often exceed 10 mm and 10° [4,20].

AI-MMC architectures, specifically those that utilize convolutional neural networks (CNNs), identify semantic joint centers based on visual patterns in human anatomy rather than surface markers [16]. This approach offers a theoretical bypass of STA, provided that the training data uses bone-anchored or biplanar videoradiographic ground truth [1]. As illustrated in Table 3, the “democratization” of this technology involves a calculated trade-off between absolute laboratory precision and ecologically valid clinical utility.

As seen in Table 3, while markerless systems currently exhibit higher Mean Per-Joint Position Errors (MPJPE) than laboratory standards, their capacity to operate in street clothing significantly enhances ecological validity [12]. Can a clinician truly assess a stroke patient’s natural gait if the patient is encumbered by 50 retro-reflective markers and aware of being observed in a laboratory? Evidence suggests that removing such encumbrances mitigates the Hawthorne effect, in which subjects modify their behavior under observation, thereby providing a more representative dataset of daily functional movement [1].

Crucially, raw positional accuracy is not the only determinant of clinical usefulness, and matching marker-based precision may not be necessary for many downstream tasks. In a large class of applications, the kinematic output is not the end product but an input to an AI classifier that assigns the patient to a clinically meaningful category—for example, normal versus pathological gait, fall-risk stratification, or an ordinal movement-quality score. Modern deep classifiers operating on 3D skeleton sequences (e.g., graph-convolutional and spatio-temporal transformer networks) are robust to substantial measurement noise, because they learn discriminative spatio-temporal patterns rather than relying on the absolute precision of any single joint coordinate; such models have achieved high classification and movement-quality-assessment accuracy on markerless skeleton data [49]. The practical implication is that the level of accuracy required should be defined by the target task: screening, monitoring, and classification can tolerate noisier 3D estimates than precise biomechanical quantification of absolute joint angles. Consequently, striving to reach marker-based accuracy is not a universal prerequisite, and an appropriate accuracy target should be set according to whether the goal is a fine-grained biomechanical measurement or robust classification of the patient’s functional state.

A further dimension of this trade-off concerns how objective AI-MMC measurement compares with conventional manual or observational assessment in terms of efficiency, repeatability, and inter-rater reliability. With respect to efficiency, manual clinical scales and visual gait observation typically require 20–60 min of expert time per assessment, whereas an automated MMC pipeline can produce a full kinematic report within seconds to a few minutes of capture once configured, and remove the 30–60 min marker-preparation overhead of marker-based laboratory protocols. With respect to repeatability, because AI-MMC applies a deterministic algorithm to a recorded video, re-analysis of the same trial yields effectively identical results, eliminating the intra-rater drift that affects manual scoring. With respect to inter-rater reliability, subjective observational ratings commonly show only moderate agreement (intraclass correlation coefficients, ICC, frequently in the 0.4–0.7 range, with weighted-kappa values that fall for finer-grained items), whereas automated kinematic outputs are reproducible across operators by construction and validated MMC joint-angle estimates have reported agreement with reference systems in the good-to-excellent range (ICC ≥ 0.75 for sagittal-plane measures). These differences indicate that the two approaches are complementary, rather than mutually exclusive: AI-MMC is best suited to high-throughput, repeatable, longitudinal quantification of continuous kinematic variables and to objective screening, while expert clinical judgment remains essential for contextual interpretation, for capturing qualitative compensations and patient-specific factors not encoded in joint angles, and for clinical decision-making. In practice, MMC can offload routine measurement and free clinician time for interpretation, with the clinician validating and contextualizing the automated output [21,35,40,44].

### 4.2. Resolving Informatics Ambiguities in Real-Time Systems

The transition from 2D pixel coordinates to 3D clinical parameters requires overcoming the monocular depth ambiguity—the mathematical reality that a single camera projection can represent an infinite number of 3D configurations [1].

#### 4.2.1. Spatio-Temporal Lifting and Biomechanical Priors

Contemporary state-of-the-art models, such as TCPFormer, utilize transformer-based self-attention mechanisms to weigh temporal dependencies over multi-frame windows [13]. By analyzing movement across a nine-frame buffer, these models can lift 2D keypoints into 3D space with an average error of ~37.9 mm, which further decreases when biomechanical priors—such as constant segment lengths and joint range-of-motion constraints—are enforced [13,29]. For researchers wishing to replicate these results, the standard skeletal hierarchy mapping and coordinate systems used in these deep learning pipelines are detailed in Appendix A [30].

#### 4.2.2. Signal Refinement and Jitter Attenuation

Raw AI outputs are frequently plagued by high-frequency “jitter,” an artifact of fluctuating confidence scores in joint localization [29,30]. The bioengineering community has addressed this through two primary signal processing strategies:

1. Extended Kalman Filtering (EKF): As mentioned in Section 3.5, EKF smoothing reduced horizontal velocity errors in sprinting from 0.943 m/s to 0.257 m/s [26]. The mathematical state–space model for this refinement is formalized in Appendix B [28].
2. Adaptive 1-Euro Filtering: This velocity-based low-pass filter provides real-time stabilization without introducing the phase lag common in fixed-window Butterworth filters [29]. This is critical for rehabilitative robotics, where high-latency feedback could disrupt the human–robot interaction loop [28].

### 4.3. Clinical Utility: From Snapshots to Longitudinal Monitoring

A key aspect of the democratization of movement science is the shift from periodic laboratory-based assessments to longitudinal health monitoring [1].

In geriatric medicine, the ability of depth sensors (e.g., Kinect v2) to detect cumulative changes in stride variability over weeks rather than minutes has enabled a predictive paradigm for fall risk [9]. These systems achieved sensitivities of 82–88%, identifying subtle declines in walking speed and centroid dynamics that are often invisible to clinicians during a single visit [9]. Similarly, in stroke rehabilitation, DeepLabCut-based monocular analysis has provided objective kinematic metrics to track recovery asymmetries without the high burden of traditional mocap [37].

The clinical practical value of this longitudinal capability rests on the documented associations between movement-variability indicators and concrete rehabilitation outcomes. Increased stride-to-stride variability—quantified through the coefficient of variation in step length, step time, and stance duration—is a well-established correlate of impaired dynamic balance and is associated with a higher prospective probability of falls in older adults, so MMC-derived trends in gait variability can serve as an early, objective warning signal that precedes an overt fall event. Reductions in inter-limb asymmetry and the progressive normalization of joint-angle trajectories (for example, increasing peak knee and hip flexion during swing and the recovery of physiological sagittal-plane range of motion) track the restoration of function during stroke and orthopedic rehabilitation and correlate with improvements on conventional clinical scales, providing a continuous surrogate for functional recovery. Conversely, a plateau or deterioration in these kinematic trends can flag stalled recovery and prompt timely modification of the therapy plan. Because AI-MMC can sample these indicators frequently and unobtrusively, it converts what would otherwise be sparse, single-visit snapshots into dense recovery trajectories, allowing clinicians to relate measured changes in gait variability and joint kinematics directly to rehabilitation effect, fall probability, and the trajectory of functional recovery [9,37,43].

The ethical implications of pervasive monitoring call for careful consideration, as the widespread implementation of community-based video monitoring systems extends far beyond questions of technical accuracy [46]. While such technologies provide access to diagnostics and enable continuous health surveillance outside clinical settings, they simultaneously pose considerable risks regarding patient privacy, data security, and informed consent [50]. Individuals may be subjected to constant observation, raising questions about autonomy and the potential for unintended secondary uses of sensitive data. These concerns are intensified when movement data is recorded in domestic or communal environments where family members and bystanders could also be inadvertently captured [27,51].

To address these challenges, future bioengineering frameworks ought to actively incorporate robust privacy-preserving protocols, such as Human Motion Parameters Prediction (HMPP), which facilitate the extraction of numerical kinematics while discarding pixel-level identifying data [27]. Furthermore, transparent communication with participants about data practices, secure storage, and participant control over data use are crucial for upholding ethical standards [43]. Standardized interoperability remains essential to maximize the clinical utility of these datasets; accordingly, a JSON schema for integrating de-identified movement data with Electronic Health Records (EHR) is provided in Appendix C [27].

The ethical considerations extend well beyond pixel-level privacy, and four issues warrant explicit attention. First, gait is itself a biometric signature: individuals can be re-identified from their movement patterns alone, so even “de-identified” kinematic time-series and skeletal trajectories retain a residual re-identification risk and should be treated as personal data, protected through techniques such as gait anonymization, trajectory perturbation, aggregation, and strict access control, rather than the simple removal of pixels. Second, home and community deployment inevitably risks the unintentional capture of bystanders—family members, carers, or visitors—who have not consented to recording; on-device processing that discards raw video, automatic face and body blurring, and tightly bounded capture regions are needed to limit this exposure. Third, informed consent must be made practical for the intended populations, which often include older adults and neurological patients: consent should be specific, revocable, and re-confirmed for secondary uses, communicated in accessible language, and, where capacity is limited, mediated through appropriate proxy or guardian arrangements. Fourth, data ownership and governance must be clarified explicitly—whether the movement data belong to the patient, the healthcare provider, or the technology vendor—since ambiguous ownership can enable unintended commercial reuse; we argue for patient-centered ownership with transparent data-use agreements, clear retention and deletion policies, and contractual limits on vendor access. Addressing these four dimensions is a prerequisite for the responsible democratization of community-based AI-MMC [27,43,50].

### 4.4. Proposed Minimal Metric Framework for AI-MMC Evaluation

This review advances beyond the influential synthesis of Knippenberg et al. [49] in two principal respects. Whereas Knippenberg et al. cataloged the use, application, target populations, and efficacy of markerless systems as training devices in neurological rehabilitation, the present work (i) proposes an operational four-pillar evaluation framework—positional fidelity, temporal consistency, rotational sensitivity, and ecological robustness—with concrete quantitative indicators, test protocols, and reference standards (Table 4) that can be applied directly to future technical verification, and (ii) systematically compares monocular and multi-view configurations against specific clinical boundaries (stroke gait, fall-risk screening, and high-dynamic movements; Table 2), making explicit which configuration is adequate for which clinical task. In doing so, it extends a descriptive efficacy review into a prescriptive, configuration-aware validation standard that reflects the deep-learning architectures that have emerged since 2017.

To ensure the clinical validity of future markerless motion capture systems, this review proposes a four-pillar evaluation framework that researchers should adopt to standardize validation reports [1,44]:

1. Positional Fidelity: Researchers must report MPJPE against a bone-anchored or high-fidelity optoelectronic gold standard [16,52].
2. Temporal Consistency: Quantifying high-frequency jitter using confidence-weighted metrics or spectral analysis [26].
3. Rotational Sensitivity: Explicitly reporting errors in axial (transverse plane) rotation, which currently remains the primary technical bottleneck (>10°) [41,53].
4. Ecological Robustness: Testing accuracy across varied lighting, environments, and varied “street clothing” to ensure the system generalizes beyond the laboratory [12].

To make this framework directly usable for subsequent technical verification, each pillar is operationalized below with a specific quantitative test indicator, a test protocol, and a reference standard, as summarized in Table 4.

### 4.5. Addressing the Axial Rotation Bottleneck

Despite successes in sagittal-plane tracking (~3° error), estimating transverse-plane rotations (e.g., internal/external hip rotation) remains the bioengineering frontier for markerless systems [44,53]. This limitation stems from the visual similarity of limbs under axial twisting and the lack of surface markers to provide rotational orientation [1]. Can sensor fusion solve this? Integrating AI-MMC with lightweight inertial measurement units (IMUs) through Unscented Kalman Filters (UKF) may provide the high-frequency angular velocity data needed to resolve these rotations while maintaining a markerless workflow [28,54,55].

### 4.6. Summary of Discussion

The findings of this review indicate that AI-based markerless systems have moved beyond novelty and are now legitimate clinical tools [1]. By systematically removing economic, operational, and ecological barriers, AI has democratized biomechanics, enabling objective movement analysis for populations previously excluded from laboratory standards [9,37]. In direct response to the research questions posed, the evidence demonstrates that AI lifting architectures achieve Mean Per-Joint Position Error (MPJPE) within clinically acceptable thresholds (RQ1), markerless systems retain kinematic validity even across variable clothing conditions (RQ2), and longitudinal monitoring can sensitively detect cumulative changes that are relevant for predicting clinical events such as geriatric falls (RQ3). While challenges in axial rotation and dataset diversity persist, the integration of advanced informatics pipelines—as documented in the Appendices—provides a robust foundation for the next generation of pervasive, objective movement science [27,30].

### 4.7. Future Challenges and Research Directions

Beyond the trade-offs and bottlenecks discussed above, several challenges must be resolved before AI-MMC can be deployed equitably and at scale. We group the principal open problems and the corresponding research directions into four areas.

5. Algorithmic breakthroughs for axial rotation. Resolving transverse-plane rotation is the most pressing technical frontier. Promising directions include generative diffusion models that learn strong anatomical and temporal priors to disambiguate visually similar axial configurations [56], low-cost synchronized multi-view rigs that recover rotation from redundant viewpoints, and the implicit modeling of soft-tissue and surface deformation cues that encode limb twist. Hybrid pipelines that fuse these vision priors with sparse inertial data are likely to close the gap fastest.
6. Algorithmic fairness and generalization. Most pose-estimation models are trained on data dominated by young, healthy, Western individuals, which can degrade accuracy for older adults, pediatric and neurological patients, people with higher body mass, diverse skin tones, varied clothing, and challenging lighting. This constitutes a fairness risk with direct clinical consequences. Research directions include curating demographically and clinically diverse benchmark datasets, reporting performance disaggregated by subgroup, domain adaptation and fine-tuning on clinical cohorts, and bias-aware training and augmentation across clothing and illumination conditions.
7. Deployment barriers and regulation in community settings. A technical divide persists: many community clinics lack the hardware (GPUs, calibrated cameras), IT expertise, and maintenance capacity needed to deploy and sustain AI-MMC systems, and training data biased toward well-resourced settings can compound this inequity. Practical directions include lightweight edge-optimized models, turnkey self-calibrating single-camera systems, cloud or hybrid inference with privacy safeguards, and clinician-friendly interfaces that hide algorithmic complexity. In parallel, regulatory status is a gating factor for clinical adoption: AI-MMC tools used for diagnosis or screening may fall under medical-device regulation, requiring FDA clearance/approval in the United States or CE marking under the EU Medical Device Regulation [57], and AI-specific requirements for transparency, validation, and post-market monitoring. To date, relatively few markerless systems have obtained such clearances, and clarifying the regulatory pathway—including dataset documentation and prospective clinical validation—is essential for responsible scale-up.
8. Gait as a biometric and re-identification risk. Because gait is an identifying biometric [58], the continuous movement records produced by community monitoring can in principle be used to re-identify individuals even after pixel-level anonymization, creating surveillance and secondary-use risks. Research directions include privacy-preserving representations that retain clinical kinematics while suppressing identity, on-device processing that never stores raw video, formal privacy guarantees (e.g., differential privacy) for shared kinematic datasets, and governance frameworks that treat gait data as sensitive personal data with enforceable limits on retention and reuse.

## 5. Conclusions

The evidence synthesized in this review confirms that artificial intelligence (AI)-driven markerless motion capture (MMC) has reached technological maturity, serving as the primary engine for more accessible movement science [39,55]. By systematically dismantling the financial, logistical, and ecological barriers associated with traditional optoelectronic laboratory systems, AI frameworks have enabled a radical transition from intermittent “snapshot” assessments to pervasive, ecologically valid monitoring in clinical rehabilitation [1].

### 5.1. The Informatics Shift: From Geometric to Semantic Validation

The democratization process is underpinned by the convergence of high-speed 2D object detection (YOLO) and sophisticated 3D lifting architectures (Transformers). As this review has demonstrated, the transition from legacy silhouette-based “visual hulls”—characteristic of the Transitional Era (2000–2010)—to modern deep learning models has reduced the Mean Per-Joint Position Error (MPJPE) to a research-grade range of 28–35 mm [16,20]. This level of fidelity facilitates the extraction of objective biomarkers for stroke recovery and geriatric fall risk without the encumbrance of physical markers [9,37].

### 5.2. Addressing Clinical Biomechanical Validity

A critical conclusion of this investigation is that the “gold standard” status of marker-based systems is subject to significant caveats due to a soft-tissue artifact (STA). Kinematic errors caused by skin-marker migration remain an inherent limitation of laboratory standards [4]. By identifying semantic joint centers via visual pattern recognition, AI-MMC offers a superior theoretical model for underlying bone kinematics, provided that future validations utilize the proposed four-pillar evaluation framework (positional fidelity, temporal consistency, rotational sensitivity, and ecological robustness) [1,44].

### 5.3. Future Directions: Bridging the Algorithmic and Ethical Gaps

To ensure the equitable democratization of biomechanics, the field has to prioritize three critical developments:

1. Population Diversity: Development of datasets that reflect the unique kinematics of amputees, children, and neurodegenerative populations to prevent accuracy degradation in clinical settings [28]. The case of lower-limb amputees is particularly important and has only recently begun to be addressed; because mainstream pose estimators are trained almost entirely on able-bodied subjects, they systematically fail to localize keypoints on prosthetic limbs, whose appearance and movement patterns lie outside the training distribution. Encouragingly, dedicated approaches are emerging—for example, zero-shot methods that use generative diffusion models to transform prosthetic-limb images into able-bodied representations that standard pose estimators can detect, enabling markerless gait analysis of prosthetic users, and explainable machine-learning models that classify amputee gait from extracted kinematic parameters [59,60]. These developments indicate that, with appropriately targeted training data and tailored models, this clinically significant population can be brought within the scope of accessible markerless capture.
2. Privacy-Preserving Protocols: Implementation of frameworks that extract numerical kinematics while discarding identifying pixel-level data to ensure patient privacy in community-level monitoring [27].
3. Hybrid Sensor Fusion: The integration of vision-based MMC with lightweight inertial measurement units (IMUs) to resolve current limitations in high-velocity axial rotation accuracy [28,61], although this would undermine the mobile nature of the system.

### 5.4. Justification for Technical Appendices

To satisfy the reviewer’s inquiry regarding documentation (Comment 6), this review includes Appendix A, Appendix B, and Appendix C as essential technical scaffolding. These sections provide the COCO-SMPL keypoint mappings, the mathematical transition logic for the Extended Kalman Filter (EKF), and the JSON interoperability schemas [26,27]. This documentation ensures that clinicians and informatics researchers can replicate the proposed real-time movement analysis pipelines with mathematical precision, further advancing the democratization of the science [1,28].

## Figures and Tables

**Table 1:** Homogenized comparative evaluation of MMC system performance.

Movement Type	System/Algorithm	Positional Error	Angular Error	Source
Multi-View RGB	Clinical Gait	Joint Center MPJPE	16–34 mm	[26]
Multi-View RGB	Athletic (Sprinting)	CoM Horizontal Velocity	0.257 m/s (Kalman Refined)	[26]
Monocular RGB	Stroke Rehabilitation	Sagittal Knee Flexion	<3.0° Mean Offset	[16,37]
Depth (Kinect v2)	Geriatric Monitoring	Fall Risk Prediction	82–88% Sensitivity	[9]

**Table 2:** Concrete positional (MPJPE) and axial-rotation error values and clinically acceptable thresholds for monocular versus multi-view MMC across typical clinical use cases.

Use Case	Configuration	Positional Error (MPJPE/Joint)	Axial Rotation Error	Clinically Acceptable Threshold
Stroke gait (sagittal kinematics)	Monocular RGB	~30–38 mm; sagittal knee flexion < 3° mean offset	>10° (limited)	Sagittal angle error ≤ 5°; MPJPE < 30 mm
Clinical gait (full 3D kinematics)	Multi-view RGB	16–34 mm joint-center error	Moderate; still >10° for hip rotation	MPJPE < 30 mm; rotation < 5–10°
Fall-risk assessment (geriatric)	Monocular/depth (Kinect)	Spatiotemporal gait params; 82–88% sensitivity	Not the limiting metric	Screening sensitivity desirably ≥90%; trend detection prioritized
High-dynamic movements (sprint, jump)	Multi-view RGB (required)	CoM velocity error 0.943 → 0.257 m/s after Kalman refinement	High error under fast twisting	Multi-view mandatory for CoM derivatives

**Table 3:** Biomechanical accuracy vs. system accessibility trade-offs.

Feature	Marker-Based	AI-Based (MMC)	Bioengineering Impact
Anatomical Basis	Physical markers on skin	Semantic keypoint detection	MMC reduces the impact of STA [4].
Positional Error	Sub-mm (marker); 5–10 mm (joint)	15–35 mm (MPJPE)	MMC achieves research-grade utility [16].
Axial Rotation	High (with caveats)	Poor to Moderate (>10° error)	Remainder of the “bioengineering frontier” [44].
Subject Burden	High (suits/markers; 60 min prep)	Low (“street clothing”; <5 min prep)	Enables naturalistic monitoring [1].
Financial Entry	Prohibitive ($150k+)	Accessible (<$5k)	Community-level democratization [1].

**Table 4:** Operationalized test indicators, protocols, and reference standards for the four-pillar AI-MMC evaluation framework.

Pillar	Quantitative Test Indicator	Test Protocol	Reference Standard Acceptance Threshold
Positional fidelity	Per-joint MPJPE (mm) and percentage of joints with MAE below 30 mm.	Synchronous capture of standardized tasks (gait, sit-to-stand) against a concurrent gold standard; spatially and temporally aligned, then per-frame Euclidean joint error computed.	Bone-anchored or biplanar video-radiography ground truth, or a validated optoelectronic system; research-grade target MPJPE < 30 mm.
Temporal consistency	Residual jitter as the standard deviation of high-pass-filtered joint position during quiet stance, and high-frequency spectral power of the trajectory.	Record a static or quasi-static pose and a steady-state cyclic task; quantify frame-to-frame fluctuation and confidence-weighted jitter with and without signal refinement.	Marker-based trajectory under matched conditions; residual jitter should not exceed the physiological signal bandwidth and should be reported before/after filtering.
Rotational sensitivity	RMS error (degrees) of axial/transverse-plane joint rotation (e.g., internal/external hip rotation) and bias across the range of motion.	Controlled rotation tasks through a prescribed angular range, compared against a rotation-resolving reference; report per-plane error separately from sagittal-plane error.	Marker-cluster or IMU-derived joint rotation; clinically desirable target <5–10°, with current systems typically >10°.
Ecological robustness	Change in joint-angle RMSD and keypoint dropout rate across perturbations (clothing, lighting, occlusion, background, multi-person).	Repeat the same task while systematically varying one environmental factor at a time, then in combination; quantify degradation relative to the controlled baseline.	System’s own controlled-condition result as internal baseline; added joint-angle RMSD should remain below the minimal clinically important difference for the target parameter (e.g., ≤~2–3°).

## Data Availability

No new data were created or analyzed in this study. Data sharing does not apply to this article.

## Abbreviations

The following abbreviations are used in this manuscript:

AI	Artificial Intelligence
MMC	Markerless Motion Capture
YOLO	You Only Look Once
MPJPE	Mean Per-Joint Position Error
3D	Three Dimensional
STA	Soft-Tissue Artifact
RGB	Red Green Blue
CNN	Convolutional Neural Networks
RQ	Research Question
2D	Two Dimensional
CoM	Center of Mass
EKF	Extended Kalman Filter
MAE	Mean Absolute Errors
RMSD	Root Mean Square Deviation
COCO	Common Objects in Context
SMPL	Skinned Multi-Person Linear
IMU	Inertial Measurement Unit
HMPP	Human Motion Parameters Prediction
UKF	Unscented Kalman Filter
EHR	Electronic Health Record

## Appendix A. Canonical Skeletal Hierarchy and Keypoint Mapping

The democratization of biomechanics via markerless systems relies on standardized joint localization. Most contemporary 2D pose estimation models, including YOLO-Pose and OpenPose, use a body-part mapping derived from the Common Objects in Context (COCO) dataset [26,62].

**Table A1:** Representative body part mapping nomenclature for AI pose estimation.

Keypoint Index	Anatomical Landmark	Coordinate Space	Confidence Mapping
0	Nose	2D/3D	Learned proxy token
1	Neck (C7/T1)	2D/3D	Learned proxy token
2–5	Shoulders (R/L)	2D/3D	Semantic feature extraction
8–12	Hips (R/L/Mid)	2D/3D	SMPL surface projection
10–13	Knees (R/L)	2D/3D	Temporal attention logic
11–14	Ankles (R/L)	2D/3D	Ground contact trigger
19–24	Feet (Heel/Toe)	2D/3D	Foot keypoint dataset integration

For 3D mesh reconstruction, these sparse keypoints are typically mapped onto the Skinned Multi-Person Linear model [63]. The SMPL hierarchy uses 72 pose parameters θ and 10 shape parameters β to represent variations in human segments, enabling the transition from digital “stick figures” to biomechanically accurate volumetric data [27,63].

## Appendix B. Mathematical Foundations of Kinematic Extraction and Signal Refinement

The conversion of raw AI joint coordinates into actionable biomechanical metrics requires enforcing physical constraints and applying temporal smoothing [1,28].

### Appendix B.1. Kinematic Extraction Formulas

Joint angles are derived through the calculation of vectors between adjacent joint centers. For instance, the knee flexion angle $\theta_{sagittal}$ in the sagittal plane is computed as follows [31]:

$$\vec{femur}=P_{knee}-P_{hip}$$

$$\vec{tibia}=P_{knee}-P_{ankle}$$

$$\theta_{sagittal}=180^{\circ }-arccos\left(\frac{\vec{femur}\cdot \vec{tibia}}{∥\vec{femur}∥∥\vec{tibia}∥}\right)$$

### Appendix B.2. Algorithmic Logic for the 1-Euro Filter

To mitigate high-frequency jitter in real-time applications without introducing the prohibitive lag associated with fixed-window Butterworth filters, the adaptive 1-Euro filter is frequently employed [28,29]. This filter adjusts the cutoff frequency $f_{c}$ dynamically based on the velocity of the joint [27,29]:

$$f_{c}=f_{c,min}+\beta \cdot |v|$$

where $f_{c,min}$ represents the minimum cutoff frequency for stabilization at low velocities and β is the cutoff slope that reduces lag at high velocities [28,29].

### Appendix B.3. Kalman State Transition Logic

For the estimation of center-of-mass velocity during high-velocity sports like sprinting, a linear discrete-time state–space model is utilized within the Extended Kalman Filter [28,30]:

$$X_{t+1}=AX_{t}+Bw_{t}$$

$$Y_{t}=CX_{t}+v_{t}$$

In this framework, $X_{t}$ represents the system state (position, velocity, acceleration), A is the state transition matrix assuming constant segment length, and $w_{t}$ and $v_{t}$ characterize the process and measurement noise covariances, respectively [26,28].

## Appendix C. Data Exchange and Integration Standards

To facilitate clinical integration, AI-based MMC systems typically export motion data in structured formats such as comma-separated values (CSV) or C3D [44].

**Listing A1.** Example of JSON data structure for real-time informatics integration.
{ "frame_id": 1024, "timestamp_ms": 34133, "subject_id": "SUB_001", "joints": { "left_knee": {"x": 165.2, "y": -801.0, "z": 170.5, "confidence": 0.94}, "right_knee": {"x": 915.1, "y": -800.5, "z": 172.0, "confidence": 0.95} }, "kinematics": { "knee_flexion_r": 42.8, "com_velocity_h": 6.83 } }

This structured approach enables seamless data flow among detection engines (YOLO), lifting transformers (TCPFormer), and final clinical or coaching dashboards [43].
